# Medical students’ knowledge on palliative care – a survey of teaching in Finland

**DOI:** 10.12688/mep.20013.2

**Published:** 2024-07-22

**Authors:** Leila Niemi-Murola, Aija Vanhanen, Outi Akrén, Peeter Karihtala, Reino Pöyhiä

**Affiliations:** 1 University of Helsinki, Helsinki, Finland; 2 Department of Anaesthesiology and Intensive Care Medicine, Helsinki University Hospital, Helsinki, Finland; 3 Laakso Hospital, Helsinki City Hospital, Helsinki, Finland; 4 University of Turku, Turku, Finland; 5 Palliative Centre, Turku University Hospital, Turku, Finland; 6 Department of Oncology, Helsinki University Hospital Comprehensive Cancer Centre, Helsinki, Finland; 7 University of Eastern-Finland, Kuopio, Finland; 8 Palliative Centre, Essote, Mikkeli, Finland

**Keywords:** palliative medicine, undergraduate teaching, medical students

## Abstract

**Background:**

Most studies on palliative medicine (PM) undergraduate education have focused on contents and organizational issues but not the outcome. Students’ learning outcomes should be studied to improve teaching in medical schools.

**Methods:**

A questionnaire about perceived PM education and attitudes on palliative care (PC) was sent to 543 last year students in all five Finnish medical schools in 2018–2019. In total, 175 (32 %) responses were received from four universities. The students evaluated both the quantity and quality of their PM teaching, implementation of European Association for Palliative Care (EAPC) guidelines and their satisfaction to the training. There were two palliative case scenarios, and the students were asked to find the best treatment option. In addition, students´ attitudes towards end-of-life (EOL) care issues were examined.

**Results:**

In the Finnish universities, PM education was available mainly integrated with oncology, geriatrics, and general medicine. A total of two universities also offered a specific PM course. In average, 50–70% of the EAPC curriculum was covered by lectures, small-group teaching, seminars, and bedside teaching with significant differences between faculties. Only 30–60 % of students were satisfied with the education received. The highest rankings were given in the universities with a special PM course. Students from these universities expressed less anxiety in facing EOL issues.

**Conclusions:**

In Finland, the coverage of EAPC curriculum is satisfactory, but the PM education is mainly given integrated with other specialties. The dedicated course on PM was associated with increased perceived knowledge and satisfaction of PM education. However, PM training was not associated with students’ attitudes on PC.

## Background

WHO defines palliative care as an approach that improves the quality-of-life of patients – adults and children – and their families who are facing problems associated with life-threatening illness ^1^. Successful integration of palliative care to health care systems requires systematic education on it from undergraduate to graduate levels of physicians ^2^.

Teaching palliative medicine (PM) and end-of-life care has increased in medical schools all over the world and PM has been included in the most recent lists of intended learning outcomes of medical schools ^3– 5^. However, contents and methods of teaching vary greatly ^6– 14^.

One of the most comprehensive international guidelines for undergraduate palliative medicine teaching is the recommendations of European Association for Palliative Care (EAPC) ^15^, which have been recently renewed by Mason *et al.* ^16^ These guidelines are based on a goal-directed and content specified (see Table 1) strategy including horizontal integration to other subjects in the medical school. The recommended learning methods include problem-based learning, discussion, role play, multi-professional learning, self-reflection and group discussions of difficult situations.

**Table 1.** Methods of palliative medicine teaching. Statistically significant differences between universities. * p< 0.05, ** p < 0.01.

University	Lectures	Small group	Bedside	Seminars	Independent learning
Helsinki	24.1 (17.2)	30.0 (14.9)	7.3 (7.7)	19.7 (10.3)	18.8 (11.6)
Oulu	30.6 (17.2)	19.6 (17.7) *	2.0 (6.3) **	28.3 (19.5) *	19.5 (18.6)
Tampere	22.5 (13.9)	33.7 (12.8)	11.8 (6.3)	18.8 (9.6)	13.2 (10.6)
Turku	34.5(15.5)	25.7 (11.8)	5.3 (6.4)	11.3 (10.7)	23.3 (12.1)
Average	27.9 (16.0)	27.2 (14.3)	6.6 (6.7)	19.5 (12.5)	18.7 (13.2)

Yet, rather little is known how EAPC guidelines have been implemented in different countries and how students experience the utilization of these recommendations in perceived teaching. International recommendations may be very useful internationally for developing unified educational programs, although national and even institutional adjustments are needed. Lehto *et al.* ^17^ reported that in the university of Tampere, Finland, EAPC recommendations were exceeded in the volume of teaching, which was associated with increased knowledge among students. At the time of the current study, a special course in PM is available only in two out of five medical faculties in Finland ^18^. Unfortunately, data from other Nordic universities are lacking.

An extensive review found 126 studies about undergraduate palliative medicine education but failed to provide evidence on defining any method of teaching better than other ^13, 19– 23^. However, several studies have shown short-term benefits of a dedicated palliative care course on medical students´ improved knowledge and skills in palliative care ^24^. Yet long-term learning outcomes after palliative care education have not been systematically studied. In addition, there seems to be controversial information available, how students´ attitudes on EOL issues change during their studies. Elder studies ^25, 26^ have failed to show any impact of palliative care teaching on students’ attitudes, while more recent studies ^27– 30^ have suggested, that the students’ attitudes towards EOL care improve after well-executed palliative care education. According to several studies, visit to a hospice, interfacing a dying patient and personal exposure to death are associated with positive change in the attitudes of medical students ^31– 34^.

We think that knowledge, skills and attitudes of students in palliative care should be studied as long-term outcome ^31, 34^. Without continuous exploring of the students´ experiences this would obviously not be possible. This is why we have undertaken this study in order explore Finnish medical students’ present learning experiences and attitudes on palliative care at the end of their undergraduate education. Another aim of our study was to facilitate the development of undergraduate palliative medicine learning curriculum in Finland.

## Methods

The study was approved by the University of Helsinki Ethical review board in humanities and social and behavioural sciences (permit number 53/2017 dated March 24, 2017). All sixth (final) year medical students who attended a mandatory seminar or examination were handed an information sheet regarding the study and the questionnaire form ^35^. Students were informed that participation in the study or deciding not to participate would not impact their course evaluation. Filling out the questionnaire acted as giving consent. Written consent was not obtained due to protecting all participants’ anonymity. The University of Helsinki Ethical review board approved of this method of obtaining consent. Final year students were selected because they were close to graduating. A structured questionnaire was delivered to all five Finnish universities with medical schools. The questionnaire is based on the EAPC curriculum focusing on students’ attitudes towards palliative medicine, as well as their perceptions of the teaching they had received on the subject. The questionnaire in a paper form was given to 543 adult students during 2018–2019.

### General structure of the questionnaire

The questionnaire was developed by the authors. It contained demographic questions about the students’ age, gender, experience of cancer in the family, a possible loss of a loved one and participation in the care of a dying person, how the students had received palliative medicine tuition (in a defined special course or as integrated with other teaching), availability and participation of advanced education and research in palliative medicine. Students were also asked to estimate how the teaching had covered the EAPC curriculum, and if they were satisfied with the education, or they needed more teaching. EAPC curriculum was attached as an English original version with the Finnish questionnaire.

### Clinical case scenarios

To assess the perceived skills, the students were asked to choose the best options for care of two patients. The case scenarios were modified from examples previously published by Lehto *et al.* (2017) ^17^. The first case was a man with severe end-stage COPD, suffering from severe episodes of breathlessness despite of maximal COPD-medications and rehabilitation. He can walk only a few meters indoors. His oxygen saturation is 95% on room air and carbon dioxide levels in blood have been normal. What is the best option for reliving breathlessness: a) initiation of palliative oxygen therapy, b) small-dose opioid ( *e.g.*, morphine), c) regular corticosteroid orally, or d) small-dose benzodiazepine ( *e.g.*, lorazepam). The other case presented a 48-year-old man suffering of a pancreatic cancer with liver and lung metastases. While on paracetamol 1 g twice a day, he has severe constant pain (VAS 7/10). Which one is the primary way to improve the pain medication: a) a combination of paracetamol and codeine, b) ibuprofen 600 mg thrice a day, c) transdermal buprenorphine, d) long-acting morphine 30 mg twice a day and short-acting morphine 10 mg prn breakthrough pain or e) short-acting morphine 10 mg twice a day.

### Attitudes

We have developed an 11-item questionnaire focusing on students´ attitudes and knowledge on end-of-life care issues. The students were asked judge the items on EOL care using a 7-point Likert scale for each item (1 = fully disagree, 4 = not disagree/not agree, 7 = fully agree). We based this instrument partly a previously published English questionnaire ^34^ and have tested it in an unpublished study in Finnish language previously. Using a factorial analysis we have shown good reliability and validity of this questionnaire (see Supplement 1).

### Statistical analysis

Mean absolute values (with SD) or percentages (SD or ranges) of responses are given. One-way ANOVA followed by Scheffe’s post-hoc test was used to detect statistically significant (p <0.05) differences between the universities. Pearson’ correlation coefficients were calculated when appropriate. SPSS (SPSS 22; SPSS, Inc., Chicago, IL) and PSPP (GNU PSPP Statistical Analysis Software Release 1.4.1-g79ad47, (C) 2007 Free Software Foundation, Inc.) statistical programs were used for statistical analysis.

## Results

A total of 175 medical students, (mean age 27 ± 3.6 years) from four out of five universities returned a filled questionnaire ^36^. The gender distribution included 102 females, 67 males and six undefined gender with no differences between the universities. The mean response rate in all universities was 32 % (26–48 %). From the University of Eastern Finland (Kuopio), only four incompletely filled questionnaires were received, and were excluded from the analysis. A total of 37 % of the respondents had a family member with cancer, 68 % had experienced death of a loved one and 81 % had been involved with care of a dying patient, with no differences between the universities.

### Palliative medicine education

Table 1 presents how palliative medicine teaching was provided in the four universities. A special course of PM was available as optional in Tampere and as mandatory in Helsinki. In all universities, PM topics were also integrated with several other disciplines, of which an average of 80 % of students ranked oncology, 9 % geriatrics and 8 % general medicine as the most important ones for palliative medicine tuition ( Table 2). Other but less frequent disciplines listed for integrative PM teaching were anaesthesiology (Turku), anaesthesiology (Oulu) and pulmonology (Helsinki, Oulu, Turku). Drugs used in palliation had been taught in pharmacology in all universities. In Helsinki, Tampere and Turku some palliative tuition was received in neurology, in Oulu in psychiatry, and in Turku in medical ethics.

**Table 2.** Students’ ranking order of disciplines responsible for the most significant amount of palliative medicine teaching.

University	I	II	III	V
Helsinki	Special mandatory palliative course	Oncology	Geriatrics	General medicine
Oulu	Oncology	Geriatrics	General medicine	Miscellaneous
Tampere	Special voluntary palliative course	Oncology	Geriatrics	General medicine
Turku	Oncology	Geriatrics	General medicine	Miscellaneous

In average, 39 % (18–81 %) of the students had been offered advanced learning in PM, but only 2 % of the students in the University of Helsinki, and 15 % of those in the University of Tampere participated in the additional optional teaching. An average of 10 % (4–23 %) had been recruited to PM research, but only one student had started academic research in the University of Tampere.

### EAPC curriculum coverage

Table 3 gives an outline of the students’ assessment of how the main headings of the curriculum were covered in their tuition. Although only 36–60 % of students were satisfied with the teaching received, the most students did not see the need for more training. Only 20–28 % of students wished more education in physiotherapeutic and psychological management of pain, driving ability while using opioids, management of neuropsychiatric symptoms, anorexia, cachexia, fatigue and dermatological symptoms, psychosocial and spiritual issues, ethics including euthanasia, or continuous infusion of opioids.

**Table 3.** The coverage of EAPC guidelines in palliative medicine teaching in four Finnish universities. Figures represents average (SD) percentages of students in four Finnish universities, who had received tuition about the subject (Yes) and were satisfied with the teaching (Satisfied). Statistically significant differences were observed between Helsinki and Oulu and Turku (^*p <0.01), and Tampere *vs*. Turku *vs*. Oulu ( ^abx^ p <0.01).

Content	Helsinki		Oulu		Tampere		Turku	
	**Yes**	**Satisfied**	**Yes**	**Satisified**	**Yes**	**Satisfied**	**Yes**	**Satisfied**
Basic palliative care	40–94	40–71	26-84	22–66	33-93	44–89	16-79	25–70
Symptom control								
a. Basic	57–89	37–60	44–76	26–56	48–93	41–83	48–78	30–51
b. Pain	51–91	14–71	4–74	0–56	26–89	11–85	32–78	5–52
c. Gastrointestinal symptom management	91–94	63–74	68–72	42–50	78–81	70–78	76–79	52–57
d. Pulmonary symptom management	83–94	57–66	34–70	16–42	70–81	63–70	60–78	33–44
e. Neuropsychiatric symptom management	69	40	36	12	52	41	51	25
f. Anorexia, cachexia, fatigue	83	54	58	28	81	67	73	41
g. Thirst, dry mouth	94	69	54	32	81	81	70	49
h. Dermatological symptoms	57	28	54	34	48	48	30	24
Psychosocial and spiritual aspects	54–86	40–63	16–80	10–49	52–74	48–74	0–70	14–59
Ethical and legal issues	40–97	26–66	12–70	4–30	37–79	30–67	13–79	13–48
Communication	69	46	50	20	70	67	63	29
Team work	66	37	32	16	60	57	40	24
Mean	73.2 ^^*^	50.0 ^a^	49.6 *	30.5 ^ab^	67.0 x	60.2 ^b^	58.6 ^^x^	36.2 ^b^
SD	19.9	14.3	21.8	16.7	19.2	18.3	20.0	13.0

### Skills

The answers the students gave to the clinical case scenarios are given in Table 4. The most students made the right choices and the differences between the universities were mostly not statistically significant.

**Table 4.** Students’ answers to the clinical cases (a–d, see the Methods section). The correct answers were b for case 1 and d for case 2. Numbers (%) of responses to clinical cases 1 and 2 are presented below. Statistically significant difference between Tampere and other universities is marked with * p <0.05. NA = no answer.

University	Answers to clinical case 1					Answers to clinical case 2					
	a	b	c	d	NA	a	b	c	d	e	NA
Helsinki	11 (31)	24 (69)	0	0	0	1 (3)	0	2 (6)	32 (91)	0	0
Oulu	10 (20)	32 (64)	5 (10)	3 (6)	0	1 (2)	3 (6)	6 (12)	39 (78)	1 (2)	0
Tampere	0 *	22 (81)	2 (7)	3 (12)	0	0	0	1 (4)	24 (89)	2 (7)	0
Turku	16 (25)	34 (54)	6 (10)	2 (3)	5 (8)	0	0	2 (3)	55 (87)	1 (2)	5 (8)

### Attitudes

The students in University of Oulu experienced significantly more anxiety about the issues related to the end-of-life care than those in other universities ( Table 5).

**Table 5.** Mean ratings of the students from different universities, ANOVA: x* < 0.05 Scheffe. We’d like to ask You to answer the following questions on a scale from 1 to 7, selecting the option that describes your own experience the best. Mark (x) where appropriate.

Items	Tampere	Oulu	Helsinki	Turku
The thought of encountering a dying patient’s sorrow makes me feel anxious.	3,7 (1,4)	4,1 (1,6)	3,7 (1,7)	3,8 (1,5)
I try to avoid participating in making decisions about transfer to end-of-life care.	2,9 (1,1)	3,0 (1,6)	2,5 (1,3)	2,3 (1,1)
I would like to discuss encountering death with a more experienced colleague.	4,2 (1,5) ^x^	5,2 (1,6) * ^x^	4,8 (1,6)	4,1 (1,6)4,2 (1,5) *
I feel anxious when I encounter a suffering person	4,2 (1,6)	4,4 (1,7) *	4,4 (1,7)	4,1 (1,5) *
The thought of encountering a dying patient makes me feel anxious.	3,8 (1,3)	3,9 (1,8)	3,7 (1,6)	3,6 (1,6)
It is important for me to talk to the patient before making the “do-not-attempt-to-resuscitate” decision.	6,0 (1,3)	6,2 (0,9)	6,1 (0,8)	5,9 (1,4)
It is important for me talk to the next of kin before making the “do-not-attempt-to-resuscitate” decision for a patient.	5,7 (1,0)	5,7 (1,2)	5,6 (0,9)	5,2 (1,4)
Treating a dying patient makes me feel depressed.	3,2 (1,2)	3,8 (1,6)	3,6 (1,6)	3,2 (1,5)
I feel guilt, if my patient dies.	2,4 (1,6)	2,9 (1,7)	2,7 (1,5)	2,2 (1,2)
I believe that my education will prepare/ has prepared me to treat a patient’s suffering.	5,6 (1,3)	5,0 (1,3)	5,6 (1,0)	5,4 (1,3)
I believe that my education will prepare/ has prepared me to take care of a dying patient.	5,3 (1,3)	4,8 (1,5)	5,3 (1,3)	5,1 (1,3)

## Discussion

In Finland, PM teaching is integrated mostly to oncology in all universities and only two of them offered a special palliative medicine course. The palliative tuition covered 50–73 % of the EAPC recommendations, being highest in the universities with a dedicated course. In addition, the increased amount of perceived PM education was associated with reduced anxiety towards EOL care issues among the students.

So far, no large-scale studies have been published about the role of EAPC recommendations in students´ learning experiences. We explored students´ knowledge, skills and attitudes according to Kirkpatrick’s four-level evaluation model of training ^37, 38^. Assessment of knowledge was based on students´ recognition of content of EAPC guidelines ^15^. Amount of realized knowledge was significantly associated with special courses on PM, but still even 50–60 % students without a possibility to attend such, also recognized the EAPC curriculum as well covered during their studies. There is still room for criticism for the EAPC recommendations. The aim of undergraduate education is to provide basic capabilities for working as a primary care physician. Specific teaching and assessment should be based on predefined goals, which were only recently elaborated during the renewal of EAPC guidelines ^16^. The revised EAPC curriculum awaits to be tested in practice, yet rather similar development of curriculum has been done in Canada with good results ^39^.

Our observations are in line with previous studies, showing that a dedicated PM course increases students’ knowledge about care ^24, 28^. We used rather similar approach as did Pieters *et al.* ^40^, asking the students’ experiences on perceived tuition. One notable difference between the two studies is that, unlike Pieters *et al.*, we asked what medical specialities’ courses PM was taught in. We also asked the students, if there had been a dedicated PM course available to them. Surprisingly, the Dutch students were less confident in pain management than ours, but more confident on other palliative care teaching. The reason may be that Finnish palliative care teaching focuses more on pharmacology than other modalities in care.

Multidisciplinary programs including PM have gained high participation rates among students ^34^. The current study found that also in Finland, PM teaching is integrated mostly to oncology, geriatrics and general medicine, even in the two universities providing a dedicated PM course. Surprisingly, internal medicine was not reported as providing PM skills, even though increasing demand for appropriate palliative care for patients in end-stages of non-malignant diseases has been detected ^41^. Alarming observations were minimal role of bedside teaching and lack of multiprofessional education. Without a defined PM educational program, palliative tuition only in various disciplines might be sensitive to individual teachers´ interests and vary both quantitively and qualitatively ^42^. The new Finnish curriculum for undergraduate PM education has recently been developed and remains to be implemented in medical schools in Finland ^17^.

### Limitations

One limitation of this study is the relatively low response rate. Unfortunately, rather similar rates have been reported previously ^24^. Yet we received very good and precise answers and believe that the study presents a trustworthy picture of palliative medicine in Finnish universities. During the data collection palliative care teaching was minimal at the University of Eastern Finland (in Kuopio) which may account for the zero reliable responses from students there. It is also possible that the students with the most interest in palliative care have had higher than average response rates, potentially leading to the overestimation of the obtained results. As a learning outcome measure, we tested students’ knowledge on pharmacological management of cancer pain, but not other important skills, as communicative or psycho-social skills, which is a limitation, too. Unfortunately, any of internationally available comprehensive learning outcome measures on PM learning (such as Palliative care attitude and knowledge, PCAK questionnaire) ^43^ have not been translated to Finnish, so far. Our study lacks analyses of documented training curricula or interviews of teachers, which should naturally be a part of an appropriate audit ^17^.

## Conclusions

This study shows palliative medicine teaching is integrated mostly with oncology, and a special course of palliative medicine was available only in two out of five Finnish medical schools in 2019. A clear association was seen between medical students’ perceived knowledge and skills. The contents of EAPC guidelines were well acknowledged. A connection was observed between perceived knowledge, skills and attitudes in palliative care among students from different universities in Finland. Our findings can be utilized in both national and international development of PM teaching in undergraduate medical tuition.

## Data Availability

Figshare: Medical students' knowledge on palliative care - a survey of teaching in Finland (in English). https://doi.org/10.6084/m9.figshare.24632370 ^36^. The project contains the following underlying data: EN - 22-02-16. Kopio Kandit KAIKKI valmis 28.11.2021 (Raw underlying data) Figshare: Questionnaire for Finnish medical students on Palliative Care https://doi.org/10.6084/m9.figshare.24717861. The project contains the following extended data: EN - OPISK KYSELYLOMAKE 2018 final.docx (Questionnaire) Data are available under the terms of the Creative Commons Attribution 4.0 International license (CC-BY 4.0).
