# Supplementary material for: Medical students’ knowledge on palliative care – a survey of teaching in Finland
Source: MedEdPublish (2016). 2024 Nov 21;14:27. Originally published 2024 Apr 24. [Version 4] doi: 10.12688/mep.20013.4 (PMC11605172; doi:10.12688/mep.20013.4)
Supplement: Peer review history

# Reviewer report: Reviewer response for version 4

**DOI:** 10.21956/mep.22286.r40340

**Published:** 2024-12-12

**Authors:** Harry J Han

**Affiliations:** 1 Beth Israel Deaconess Medical Center, Harvard Medical School, Boston, Massachusetts, USA

This survey study of graduating Finnish medical students assessed their perceptions of and attitudes toward the palliative medicine (PM) education they received during medical school training. The authors found that a significant portion of PM education was integrated into other specialties. Additionally, students from schools with dedicated PM courses reported higher perceived knowledge of PM topics, as measured by the percentage of students self-reporting “yes” to EAPC curriculum content. This is an important study that provides Finnish medical schools with a baseline to track changes over time. However, several areas would benefit from additional clarity and detail to strengthen its contribution:

 1) **Disucssion:** I am unclear about how the authors reached the conclusion in the first paragraph of the discussion: *“In addition, the increased amount and quality of perceived PM education was associated with reduced anxiety towards EOL care issues among the students.” *In Results under *Attitudes*, the authors report increased anxiety in students from Oulu and satisfaction of EOL teaching correlated with higher ratings of Item 14, but otherwise do not present analyses examining associations between the amount or quality of PM education and anxiety levels. What specific analyses support this conclusion? Additionally, from the questionnaire data, it is not clear how the authors defined or measured perceived quality of education and quantity of PM education. Providing more detail about how the authors defined these predictor variables from their survey data would enhance understanding.

 2) **Methods and results:** The authors describe opportunities for advanced education, but the term “advanced education” needs clarification. Could the authors define what types of educational activities fall under “advanced learning in PM.” At Tampere and Helsinki, how does advanced learning differ from the special mandatory or voluntary palliative courses offered?

 3) **Methods:** For the questions estimating coverage of EAPC guidelines in curriculum, satisfaction, or desire for more teaching, can the authors clarify how these were measured? Were they assessed using binary options (e.g., yes/no, satisfied/unsatisfied), Likert scales, or numerical scores?

 4) **Table 1:** please provide a brief legend under the table title to describe what the numbers within each box represents, similar to what the authors have done in Tables 3 and 4. I believe these are average percentages and standard deviations, but this should be explicitly stated for clarity.

 5) **Table 3: **could the authors elaborate on the term “Mean” listed in the first column under “Content”? Does this refer to the mean percentage of EAPC topics covered in the curriculum or some composite mean score?

Have any limitations of the research been acknowledged?

Yes

Is the work clearly and accurately presented and does it cite the current literature?

Yes

If applicable, is the statistical analysis and its interpretation appropriate?

Yes

Are all the source data underlying the results available to ensure full reproducibility?

Yes

Is the study design appropriate and is the work technically sound?

Yes

Are the conclusions drawn adequately supported by the results?

Partly

Are sufficient details of methods and analysis provided to allow replication by others?

Yes

Reviewer Expertise:

Palliative Care Education, Medical Education, Supportive Oncology,

I confirm that I have read this submission and believe that I have an appropriate level of expertise to confirm that it is of an acceptable scientific standard, however I have significant reservations, as outlined above.

# Reviewer report: Reviewer response for version 4

**DOI:** 10.21956/mep.22286.r40264

**Published:** 2024-12-03

**Authors:** Cathy Payne

**Affiliations:** 1 European Association for Palliative Care, Vilvoorde, Flanders, Belgium

For me a significant limitation was that students were not asked if they had a family member who had received or was in need of palliative care due to a life-limiting or life-threatening illness (with examples of such diseases given such as heart failure, MND,  advanced dementia etc). A diagnosis of cancer is not an appropriate surrogate for someone in need of palliative care and indeed many cancer diagnoses are now curative. 

 Under the clinical case scenarios there is a typo. Reliving should be relieving.

Have any limitations of the research been acknowledged?

Partly

Is the work clearly and accurately presented and does it cite the current literature?

Yes

If applicable, is the statistical analysis and its interpretation appropriate?

Yes

Are all the source data underlying the results available to ensure full reproducibility?

Yes

Is the study design appropriate and is the work technically sound?

Yes

Are the conclusions drawn adequately supported by the results?

Yes

Are sufficient details of methods and analysis provided to allow replication by others?

Yes

Reviewer Expertise:

Palliative Care, Interprofessional education

I confirm that I have read this submission and believe that I have an appropriate level of expertise to confirm that it is of an acceptable scientific standard, however I have significant reservations, as outlined above.

# Reviewer report: Reviewer response for version 4

**DOI:** 10.21956/mep.22286.r40206

**Published:** 2024-11-28

**Authors:** Sarah Godfrey

**Affiliations:** 1 University of Texas Southwestern, Dallas, USA

The authors have adequately addressed the comments. Well done.

Have any limitations of the research been acknowledged?

Yes

Is the work clearly and accurately presented and does it cite the current literature?

Yes

If applicable, is the statistical analysis and its interpretation appropriate?

Yes

Are all the source data underlying the results available to ensure full reproducibility?

Yes

Is the study design appropriate and is the work technically sound?

Yes

Are the conclusions drawn adequately supported by the results?

Partly

Are sufficient details of methods and analysis provided to allow replication by others?

Yes

Reviewer Expertise:

Palliative care, cardiology, medical education

I confirm that I have read this submission and believe that I have an appropriate level of expertise to confirm that it is of an acceptable scientific standard.

# Reviewer report: Reviewer response for version 4

**DOI:** 10.21956/mep.22286.r40204

**Published:** 2024-11-22

**Authors:** Daniëlle Verstegen

**Affiliations:** 1 School of Health Professions Education, Maastricht University, Maastricht, The Netherlands

Approve of addition.

Have any limitations of the research been acknowledged?

Partly

Is the work clearly and accurately presented and does it cite the current literature?

Partly

If applicable, is the statistical analysis and its interpretation appropriate?

I cannot comment. A qualified statistician is required.

Are all the source data underlying the results available to ensure full reproducibility?

Partly

Is the study design appropriate and is the work technically sound?

Yes

Are the conclusions drawn adequately supported by the results?

Partly

Are sufficient details of methods and analysis provided to allow replication by others?

Yes

Reviewer Expertise:

Instructional design, palliative care education, undergraduate education, blended education.

I confirm that I have read this submission and believe that I have an appropriate level of expertise to confirm that it is of an acceptable scientific standard.

# Reviewer report: Reviewer response for version 3

**DOI:** 10.21956/mep.22122.r39355

**Published:** 2024-11-03

**Authors:** Sarah Godfrey

**Affiliations:** 1 University of Texas Southwestern, Dallas, USA

The authors conducted a survey of 175 Finnish medical students to assess their perception of and attitudes towards palliative medicine education, in addition to testing their responses to 2 hypothetical palliative care scenarios. This is a an important question worldwide and varies significantly by country but also by individual medical schools. Similar to surveys in other countries, many medical students were dissatisfied with their PM education, particularly in schools without dedicated PM education. This is an interesting question that adds to the medical education literature on palliative medicine teaching principles. Here are some suggestions for improvement: 

 Major: 

 1. The listing of disciplines for PM is a bit confusing. Maybe this would be better represented by expanding Table 2 to show all of the disciplines reported by each school. 

 2. Could you expand more on the attitudes? Were those associated with amount of or satisfaction with PM education (this appears to be mentioned in the discussion but not the results)? Were they associated at all with students' personal experiences with death and dying? 

 3. There are many typos/awkward phrases throughout the text. It would benefit from editing/rephrasing (see some examples below). 

 Minor:

 1. Possible typo: "Elder studies have failed..." Did you mean "older studies"? 

 2. Possible typo: "how the students had received palliative medicine tuition" ...Did you mean "palliative medicine education"? This typo appears in multiple locations. 

 3. Anesthesiology is listed twice for Turku and Oulu. Should combine.

Have any limitations of the research been acknowledged?

Yes

Is the work clearly and accurately presented and does it cite the current literature?

Yes

If applicable, is the statistical analysis and its interpretation appropriate?

Yes

Are all the source data underlying the results available to ensure full reproducibility?

Yes

Is the study design appropriate and is the work technically sound?

Yes

Are the conclusions drawn adequately supported by the results?

Partly

Are sufficient details of methods and analysis provided to allow replication by others?

Yes

Reviewer Expertise:

Palliative care, cardiology, medical education

I confirm that I have read this submission and believe that I have an appropriate level of expertise to state that I do not consider it to be of an acceptable scientific standard, for reasons outlined above.

# Response

**Published:** 2024-11-20

**Authors:** Aija Vanhanen

**Affiliations:** Laakso Hospital, Helsinki, Uusimaa, Finland

**Reviewer's comments:**

 The authors conducted a survey of 175 Finnish medical students to assess their perception of and attitudes towards palliative medicine education, in addition to testing their responses to 2 hypothetical palliative care scenarios. This is an important question worldwide and varies significantly by country but also by individual medical schools. Similar to surveys in other countries, many medical students were dissatisfied with their PM education, particularly in schools without dedicated PM education. This is an interesting question that adds to the medical education literature on palliative medicine teaching principles. Here are some suggestions for improvement: 

**Major: **

1. **Reviewer's comments: **The listing of disciplines for PM is a bit confusing. Maybe this would be better represented by expanding Table 2 to show all of the disciplines reported by each school. 
**Response:** The table 2 shows merely the students’ choices but not what was found in the written curriculum nor in teachers’ reports, which we did not examine. We apologize our inaccurate expressions and have underlined this concept in the revised results section in the following way in the Results, on page 4, paragraph 1:
  “According to the students, in all universities, PM topics were also integrated with several other disciplines, of which…”
2. **Reviewer's comments: **Could you expand more on the attitudes? Were those associated with amount of or satisfaction with PM education (this appears to be mentioned in the discussion but not the results)? Were they associated at all with students' personal experiences with death and dying? 
**Response: **We thank the Reviewer for rising this important issue of putative explanations for the variance in the students’ attitudes. 
  First, we wish to point out that in the table 3, the coverage of EAPC, i.e. the “amount of palliative medicine teaching” is smallest in the University of Oulu with a mean 60 % mean coverage of EAPC curriculum (compared with 60-70 % in other universities). The students from this university expressed slightly more anxiety when encountering a suffering patient and would like to discuss encountering death with a more experienced colleague, which could mean that after perceived PM education these students still felt uncertain and distressed in the care of a dying patient. We think that these observations allow us to make a conclusion of the relationship of a quantity of teaching and attitudes.  Our questionnaire included a separate item about general satisfaction of the students on perceived teaching in EOL care. This was used as an internal control for reliability of the main questionnaire on attitudes. As suggested by the Reviewer, we have now compared of the students´ ratings of their satisfaction and attitudes. In correlation analysis we found a positive correlation between the satisfaction rating of perceived teaching of end-of-life care and item #4. The following notification has been added in the revision: 
  In all universities, students’ expressed satisfaction to end-of-life care teaching correlated (r = 0,39) with high ratings of the item "14. I believe that my education will prepare/ has prepared me to take care of a dying patient." in the attitude questionnaire. The students’ previous exposure to dying did not correlate with their attitudes. We were surprised that the students’ personal experience of death among their closest ones and taking care of a dying patient did not correlate with any item in the attitude questionnaire. This seems to be opposite to many other studies ( Tong J, et al. Factors influencing death attitudes of medical students: a scoping review. Front. Public Health 2024, 12:1342800. doi:10.3389/fpubh.2024.1342800. The reason this difference may be on very patchy answers of our students: unfortunately, the students answered to many questions incompletely in our study. 
  Yet we have discussed our findings in the revised Discussion section as follows:
  We did not find any correlations between the students’ previous exposure to dying and suffering among their closest ones and their attitudes on EOL-care. This observation disagrees with some recent studies, in which students’ positive attitudes on EOL care are associated with their family experiences of dying (Ke YY, Chen DX. Analysis of death attitude of 956 medical students in vocational
  college and its inﬂuencing factors. Chinese General Practice Nursing. (2021) 19:2393–7. doi:10.12104/j.issn.1674-4784.2021.17.024; Limpawattana P, Srinonprasert V , Manjavong M et al. Thai medical students’ attitudes regarding what constitutes a “good death”: a multicenter study. BMC Medical Education. BMC Medical Education (2019) 19:78). However, quite opposite findings also have been published (Tong J, Liu Q, Liu Y, Li J, Zhang Q, Shi H. Factors inﬂuencing death
attitudes of medical students: a scoping review.  Front. Public Health 2024: 12:1342800. doi: 10.3389/fpubh.2024.1342800). We think that an exposure on dying may also be a devastating experience without a possibility to reflect and discuss about one’s feelings, which PM education can provide.
  Although it would be very important to find more reasons for attitudes, we are afraid, that our data does not allow us to make deeper speculations for two other following reasons: 1) we did not plan this study to examine attitudes, but to explore the students’ views about the perceived teaching and learning of palliative medicine, and 2) we were not able to assess the students´ attitudes before and after the palliative medicine course/teaching. Finally, our data does not suit to any qualitative analysis, which would also be a good option for studying attitudes of students.
  Ho CY et al. The impact of death and dying on the personhood of medical students: a systematic scoping review. BMC Medical Education (2020) 20:516. https://doi.org/10.1186/s12909-020-02411-y
3. **Reviewer's comments: **There are many typos/awkward phrases throughout the text. It would benefit from editing/rephrasing (see some examples below).

**Minor:**

 We thank the Reviewer for pointing out these mistakes. All suggested changes have been made in the final version of the article. 

1. Possible typo: "Elder studies have failed..." Did you mean "older studies"? 
2. Possible typo: "how the students had received palliative medicine tuition" ...Did you mean "palliative medicine education"? This typo appears in multiple locations. 
3. Anesthesiology is listed twice for Turku and Oulu. Should combine.

- Is the work clearly and accurately presented, and does it cite the current literature?
  Yes
- Is the study design appropriate and is the work technically sound?
  Yes
- Are sufficient details of methods and analysis provided to allow replication by others?
  Yes
- If applicable, is the statistical analysis and its interpretation appropriate?
  Yes
- Have any limitations of the research been acknowledged?
  Yes
- Are all the source data underlying the results available to ensure full reproducibility?
  Yes
- Are the conclusions drawn adequately supported by the results?
  Partly

____________________________________

 Competing Interests

 No competing interests were disclosed.

 ________________________________________

 Reviewer Expertise

 Palliative care, cardiology, medical education

# Reviewer report: Reviewer response for version 3

**DOI:** 10.21956/mep.22122.r39246

**Published:** 2024-10-13

**Authors:** Daniëlle Verstegen

**Affiliations:** 1 School of Health Professions Education, Maastricht University, Maastricht, The Netherlands

Reapproved after the authors did some small corrections on referencing

Have any limitations of the research been acknowledged?

Partly

Is the work clearly and accurately presented and does it cite the current literature?

Partly

If applicable, is the statistical analysis and its interpretation appropriate?

I cannot comment. A qualified statistician is required.

Are all the source data underlying the results available to ensure full reproducibility?

Partly

Is the study design appropriate and is the work technically sound?

Yes

Are the conclusions drawn adequately supported by the results?

Partly

Are sufficient details of methods and analysis provided to allow replication by others?

Yes

Reviewer Expertise:

Instructional design, palliative care education, undergraduate education, blended education.

I confirm that I have read this submission and believe that I have an appropriate level of expertise to confirm that it is of an acceptable scientific standard.

# Reviewer report: Reviewer response for version 2

**DOI:** 10.21956/mep.21981.r38057

**Published:** 2024-08-26

**Authors:** Daniëlle Verstegen

**Affiliations:** 1 School of Health Professions Education, Maastricht University, Maastricht, The Netherlands

Dear authors,

 Thank you for this new version. You have addressed my concerns in rewording the research aims, conclusions and limitations, and related it all to previous literature.

 I agree with you that there it is not clear what would be the best way to teach about palliative care, and a combination of appraoches is likely to work best.

 There is one sentence in your conclusions that I still have questions about: 'Our observations are in line with previous studies, showing that a dedicated PM course increases students’ knowledge about care'. It would help if you could add one sentence to explain what you conclude this from, as you previously explain that only two of the five participating universities have such a course.

 Thank you for adding to our knowledge on this important topic!

 Daniëlle Verstegen

Have any limitations of the research been acknowledged?

Partly

Is the work clearly and accurately presented and does it cite the current literature?

Partly

If applicable, is the statistical analysis and its interpretation appropriate?

I cannot comment. A qualified statistician is required.

Are all the source data underlying the results available to ensure full reproducibility?

Partly

Is the study design appropriate and is the work technically sound?

Yes

Are the conclusions drawn adequately supported by the results?

Partly

Are sufficient details of methods and analysis provided to allow replication by others?

Yes

Reviewer Expertise:

Instructional design, palliative care education, undergraduate education, blended education.

I confirm that I have read this submission and believe that I have an appropriate level of expertise to confirm that it is of an acceptable scientific standard.

# Reviewer report: Reviewer response for version 1

**DOI:** 10.21956/mep.21430.r37114

**Published:** 2024-06-07

**Authors:** Daniëlle Verstegen

**Affiliations:** 1 School of Health Professions Education, Maastricht University, Maastricht, The Netherlands

I am very happy to see more research about palliative care education in undergraduate medicine. Certainly an important topic that needs our attention, and starting with gathering students’ experiences and perceptions is an appropriate start. This study is a valuable addition to our knowledge base.

 I have some reservations, though:

 1. The authors start off arguing that there is a need to measure outcomes of palliative care education. However, their study is mostly focused on students’ perceptions. These are also important to research (as a first step), but then the authors cannot claim to be measuring outcomes. In the introduction this could be easily adjusted with somewhat more nuanced phrasing. In the discussion, this would need larger changes. The authors really cannot claim that they have explored students´ knowledge, skills and attitudes according to Kirkpatrick’s four-level evaluation. As far as I understand from the methods description students have been asked to indicate the real and desired coverage of EAPC guidelines. This is not an assessment of their knowledge, but could maybe be seen as an indication of it (already stretching it, but ok). I see that a previous reviewer has already given other suggestions of studies that have (partly) measured outcomes in different ways

 2. The questionnaire does have two multiple-choice questions regarding after a short case description. However, only two multiple-choice questions do also not warrant the claim that the questionnaire ‘measures’ learning outcomes. Moreover, the two questions are solely focused on the somatic aspect of palliative care (symptom management) and ignore the other three aspects of psychological, social and spiritual care which equally important in palliative care.

 3. Given these considerations I conclude that the present study is in many ways similar to previous studies, though more focused on the Finnish context and the (newest) EAPC guidelines. It certainly adds to our knowledge, but I would have expected the discussion to address similarities and dissimilarities with previous findings of, for instance, Weber et al. One surprising finding for me was that Finnish students in this study did not feel that they needed more education. This is very different from what we found in Dutch students: Pieters et al.: https://pubmed.ncbi.nlm.nih.gov/31455326/

 4. The first two sentences of the discussion seem to suggest that the author believe that a separate course about palliative care would be the optimal solution. I understand that a separate course can help to give the topic importance and that it can draw students’ attention to this important area of care. I am not opposed to the idea. However, I would argue that from a learning point of view it would probably be a better idea to integrate palliative care in many places in the curriculum and in different contexts/tasks where it could be relevant. This would enable authentic learning and stimulate transfer to practice. And the repeated attention for palliative care could also help to consolidate and gradually move to more complex tasks. There are also drawbacks to this approach, e.g. it is more difficult to organize and manage. This discussion of different options to integrate palliative care in curricula would have been interesting for the discussion.

Have any limitations of the research been acknowledged?

Partly

Is the work clearly and accurately presented and does it cite the current literature?

Partly

If applicable, is the statistical analysis and its interpretation appropriate?

I cannot comment. A qualified statistician is required.

Are all the source data underlying the results available to ensure full reproducibility?

Partly

Is the study design appropriate and is the work technically sound?

Yes

Are the conclusions drawn adequately supported by the results?

Partly

Are sufficient details of methods and analysis provided to allow replication by others?

Yes

Reviewer Expertise:

Instructional design, palliative care education, undergraduate education, blended education.

I confirm that I have read this submission and believe that I have an appropriate level of expertise to confirm that it is of an acceptable scientific standard, however I have significant reservations, as outlined above.

## References

[1] : Palliative care education in the undergraduate medical curricula: students' views on the importance of, their confidence in, and knowledge of palliative care. *BMC Palliat Care* .2019;18(1) : 10.1186/s12904-019-0458-x 72 10.1186/s12904-019-0458-x 31455326 PMC6712798

# Response

**Published:** 2024-07-05

**Authors:** Aija Vanhanen

**Affiliations:** Laakso Hospital, Helsinki, Uusimaa, Finland

Thank you for these comments and suggestions for improvement.

 1. The authors start off arguing that there is a need to measure outcomes of palliative care education. However, their study is mostly focused on students’ perceptions. These are also important to research (as a first step), but then the authors cannot claim to be measuring outcomes. In the introduction this could be easily adjusted with somewhat more nuanced phrasing. In the discussion, this would need larger changes. The authors really cannot claim that they have explored students´ knowledge, skills and attitudes according to Kirkpatrick’s four-level evaluation. As far as I understand from the methods description students have been asked to indicate the real and desired coverage of EAPC guidelines. This is not an assessment of their knowledge, but could maybe be seen as an indication of it (already stretching it, but ok). I see that a previous reviewer has already given other suggestions of studies that have (partly) measured outcomes in different ways.

 Answer: We are grateful for this excellent remark. The data was collected after the course was over, and the students had already passed their examination. It is true that we have focused on indications of the knowledge. We did also explore the attitudes of the students on EOL care issues using a Finnish questionnaire, which we have developed over long period of time. We have now included this tool both in the methods and in the results. Thus, the Kirkpatrick´s model has been at least partly followed. Because this tool has not been used in other countries, we did not include it in the original MS. However, as requested kindly by both of the Reviewers, we decided to include our instrument in this MS. We also executed a factorial analysis for our tool, and wish to include it as a supplemental material. Among our students, the short attitude-questionnaire shows a very high reliability.

 2. The questionnaire does have two multiple-choice questions regarding after a short case description. However, only two multiple-choice questions do also not warrant the claim that the questionnaire ‘measures’ learning outcomes. Moreover, the two questions are solely focused on the somatic aspect of palliative care (symptom management) and ignore the other three aspects of psychological, social and spiritual care which equally important in palliative care. Answer: There were MCQs included in the test scenarios, but it is true that the questions were focused on the somatic symptoms. It is true that the other aspects are equally important, but management of cancer pain is an essential part of palliative care, and, in Finland, it is part of the undergraduate core curriculum. We have an undergoing research project focusing on psychosocial issues of palliative care teaching and learning.

 3. Given these considerations I conclude that the present study is in many ways similar to previous studies, though more focused on the Finnish context and the (newest) EAPC guidelines. It certainly adds to our knowledge, but I would have expected the discussion to address similarities and dissimilarities with previous findings of, for instance, Weber et al. One surprising finding for me was that Finnish students in this study did not feel that they needed more education. This is very different from what we found in Dutch students: Pieters et al.: https://pubmed.ncbi.nlm.nih.gov/31455326/

 Answer: The discussion of similarities and dissimilarities has been added to the discussion (page 15, line 19 to p.16, line 3). We were also surprised to observe that the Finnish students expressed no need for further education about palliative care. This might be explained by our previous finding about the perceived anxiety about meeting a suffering patient and management of end-of-life-care (Niemi-Murola L, Nieminen JT, Kalso E, Pöyhiä R. Medical undergraduate students' beliefs and attitudes toward pain: how do they mature?. *Eur J Pain*. 2007;11(6):700-706). In addition, we may also speculate, that another reason is that the role of academic palliative medicine is still very weak and remains to be recognized better in medical education.

 4. The first two sentences of the discussion seem to suggest that the author believe that a separate course about palliative care would be the optimal solution. I understand that a separate course can help to give the topic importance and that it can draw students’ attention to this important area of care. I am not opposed to the idea. However, I would argue that from a learning point of view it would probably be a better idea to integrate palliative care in many places in the curriculum and in different contexts/tasks where it could be relevant. This would enable authentic learning and stimulate transfer to practice. And the repeated attention for palliative care could also help to consolidate and gradually move to more complex tasks. There are also drawbacks to this approach, e.g. it is more difficult to organize and manage. This discussion of different options to integrate palliative care in curricula would have been interesting for the discussion.

 Answer: At present, palliative care education is indeed integrated into other relevant courses in undergraduate education. However, the clinical teachers are no experts in palliative care medicine and thus, this aspect is diluted and sometimes non-existent. The discussion of different options has been included in the text. We wish to point out that indeed in the excellent review by Fitzpatrick et al in 2017 failed to detect any superior methodology for teaching PM, so far. Also, Dr Ostgathe and colleagues claim for a dedicated PM course in their important paper (Palliative Medicine 2007; 21: 155-156). However, thereafter many papers (including the Mason et al paper on EPAC curriculum, J Palliat Med. 2020 Dec;23(12):1571-1585 and Denney-Koelsch et al. J Palliat Med. 2018 Sep;21(9):1221-1233) have advocated for benefits of integrated teaching. Most likely a combination of a dedicated course and integration on PM would lead to the best learning.

# Reviewer report: Reviewer response for version 1

**DOI:** 10.21956/mep.21430.r37119

**Published:** 2024-05-27

**Authors:** Si Qi Yoong

**Affiliations:** 1 Alice Lee Centre for Nursing Studies, National University of Singapore, Singapore, Singapore

Thank you for conducting this interesting study. I find it valuable in describing the state of undergraduate palliative care education in Finland. However, some areas require additional clarification and revision.

 1. Please explain more about the EAPC education guidelines in the introduction, such as the domains; readers unfamiliar with palliative care may not know them. What do the authors mean by “EAPC recommendations were exceeded”?

 2. The authors claim in the abstract background and in the 4 ^th^ paragraph of the introduction that none/few studies examine the outcome of palliative medicine undergraduate education. However, in recent years, there have been many studies on this such as simulation-based learning, and this can be found easily on Google Scholar e.g.

- Thyson, T., Schallenburger, M., Scherg, A. et al. Communication in the face of death and dying - how does the encounter with death influence the patient management competence of medical students? An outcome-evaluation. BMC Med Educ 22, 25 (2022). https://doi.org/10.1186/s12909-021-03060-5
- Boland, J. W., Brown, M. E. L., Duenas, A., Finn, G. M., & Gibbins, J. (2020). How effective is undergraduate palliative care teaching for medical students? A systematic literature review. BMJ open, 10(9), e036458. https://doi.org/10.1136/bmjopen-2019-036458
- Day, Frank C. MD, MPH; Srinivasan, Malathi MD; Der-Martirosian, Claudia PhD; Griffin, Erin PhD; Hoffman, Jerome R. MA, MD; Wilkes, Michael S. MD, PhD. A Comparison of Web-Based and Small-Group Palliative and End-of-Life Care Curricula: A Quasi-Randomized Controlled Study at One Institution. Academic Medicine 90(3):p 331-337, March 2015. | DOI: 10.1097/ACM.0000000000000607
- Weber, M., Braun, J., & Schildmann, J. (2011). Effects of a ninety-minute teaching module for fourth-year medical students on a palliative care ward with student-patient encounter. Journal of palliative medicine, 14(8), 940–944. https://doi.org/10.1089/jpm.2011.0025

3. What kind of outcomes are the authors referring to? Also, please cite the review that found 124 studies. References 12, 20-24 do not support the first sentence in the fourth paragraph of the introduction. Reference 12 was published in 2017 and is not “recent” anymore. Furthermore, reference 12 included 126 studies, not 124.

 4. The literature gap was not well-defined. What does current research (both quantitative and qualitative) tell us about medical students’ learning experiences and attitudes on palliative care? This is the research question, and this needs to be explored more in-depth in the introduction.

 5. Methods: the information on ethics should be moved to the last paragraph of the methods section as a separate section on “ethical considerations”. The first paragraph of the methods section should instead include the study design and methodology, which has not been explained. What were the eligibility criteria?

 6. Was the questionnaire piloted or reviewed by an expert panel before being given to the students?

 7. The authors included only 2 case scenarios, which are inadequate to assess the students’ palliative care knowledge. Palliative care knowledge includes many domains, such as palliative care philosophy, symptom management, and psychosocial and spiritual care. Why did the authors not use validated questionnaires such as https://doi.org/10.1089/jpm.2013.0112 and https://link.springer.com/article/10.1186/s12904-019-0430-9?

 8. Table 3: No SD was presented, and it seems to be range in some places. Please ensure that they are in means (SD) as stated in the caption. The mean and SD seem to represent an average of all the content, and not the individual content categories. Please provide the means (SD) for each content.

 9. The question regarding whether they needed more teaching is not found in the tables.

 10. Discussion: “We explored students’ knowledge, skills, and attitudes according to Kirkpartrick’s four-level evaluation model of training.” I find that the questionnaire was inadequate in assessing students’ knowledge, skills, and attitudes. For knowledge, this was elaborated in a previous comment. What skills and attitudes are you referring to? In other palliative health professional education studies, skills can be assessed using skills assessment-based scores like OSCE, self-reported changes in communication skills, confidence in caring for the dying, and compassion competence etc. Attitudes can be measured using FATCOD and others. Please organize the questionnaire content in the methods “general structure of the questionnaire” to reflect this clearly.

 11. Conclusions: “A clear association was seen between medical students’ perceived knowledge and skills.” I do not see this being reported in the results. There was no correlation analysis conducted. Knowledge was conducted using only 2 questions. The questions do not reflect skills in palliative care but rather pharmacological management (which is knowledge).

Have any limitations of the research been acknowledged?

Yes

Is the work clearly and accurately presented and does it cite the current literature?

Partly

If applicable, is the statistical analysis and its interpretation appropriate?

Partly

Are all the source data underlying the results available to ensure full reproducibility?

Yes

Is the study design appropriate and is the work technically sound?

Yes

Are the conclusions drawn adequately supported by the results?

Partly

Are sufficient details of methods and analysis provided to allow replication by others?

Yes

Reviewer Expertise:

Palliative care education, health professions education, palliative care

I confirm that I have read this submission and believe that I have an appropriate level of expertise to confirm that it is of an acceptable scientific standard, however I have significant reservations, as outlined above.

## References

[1] : Communication in the face of death and dying - how does the encounter with death influence the patient management competence of medical students? An outcome-evaluation. *BMC Med Educ* .2022;22(1) : 10.1186/s12909-021-03060-5 25 10.1186/s12909-021-03060-5 35012542 PMC8744229

[2] : How effective is undergraduate palliative care teaching for medical students? A systematic literature review. *BMJ Open* .2020;10(9) : 10.1136/bmjopen-2019-036458 e036458 10.1136/bmjopen-2019-036458 32912945 PMC7482461

[3] : A comparison of Web-based and small-group palliative and end-of-life care curricula: a quasi-randomized controlled study at one institution. *Acad Med* .2015;90(3) : 10.1097/ACM.0000000000000607 331-7 10.1097/ACM.0000000000000607 25539518 PMC4340770

[4] : Effects of a ninety-minute teaching module for fourth-year medical students on a palliative care ward with student-patient encounter. *J Palliat Med* .2011;14(8) : 10.1089/jpm.2011.0025 940-4 10.1089/jpm.2011.0025 21767167

[5] : The palliative care knowledge questionnaire for PEACE: reliability and validity of an instrument to measure palliative care knowledge among physicians. *J Palliat Med* .2013;16(11) : 10.1089/jpm.2013.0112 1423-8 10.1089/jpm.2013.0112 24073719

[6] : Development of palliative care attitude and knowledge (PCAK) questionnaire for physicians in Kuwait. *BMC Palliat Care* .2019;18(1) : 10.1186/s12904-019-0430-9 49 10.1186/s12904-019-0430-9 31170968 PMC6555752

# Response

**Published:** 2024-07-05

**Authors:** Aija Vanhanen

**Affiliations:** Laakso Hospital, Helsinki, Uusimaa, Finland

Thank you for these comments and suggestions for improvement.

 1. Please explain more about the EAPC education guidelines in the introduction, such as the domains; readers unfamiliar with palliative care may not know them. What do the authors mean by “EAPC recommendations were exceeded”?

 Answer: Recommendations of the European Association for Palliative Care (EAPC) For the Development of Undergraduate Curricula in Palliative Medicine At European Medical Schools (14) was published in 2013. The EAPC had formed in 1988. At that time Palliative Care was taught only at few medical schools in Europe. During the years there was growing needs for both Palliative Care services and education. It was noticed that every undergraduate medical student needs to learn about Palliative Care. The Core Content of the EAPC Recommendation

**A. The content of the curriculum** should focus on seven domains of practice, in order to achieve a basic level of competencies in Palliative Care. These seven domains are integrated within the six sections of the syllabus

 1. Basics of Palliative Care

 2. Pain and symptom management

 3. Psychosocial and spiritual aspects

 4. Ethical and legal issues

 5. Communication

 6. Teamwork and self-reflection.

**B. Goals** : Achieve graduated physicians´ preparedness of attitude, knowledge and skills necessary to participate in effective and compassionate Palliative Care.

**C. Educational strategies**: Experiential learning, Active rather than passive techniques should be applied (problem based learning, discussion, role play), Multi-professional learning,  Horizontal integration, Repeated occasions for self-reflection and group discussions of difficult situations, Ethical and psychosocial considerations should be integrated into all aspects of teaching.

**D.  Syllabus** : A suggestion for the split for the percentages of the topics within the syllabus: 1. Basics of Palliative Care 5%  2. Pain and symptom management 50% 3. Psychosocial and spiritual aspects 20% 4. Ethical and legal issues 5% 5. Communication 15% 6. Teamwork and self-reflection 5%. We have added these clarifications to the text (fourth paragraph of the introduction). We have provided a deeper description of this curriculum in the introduction. Please, notify, that the content is already described in the Table 3. which presents the coverage of EAPC guidelines in palliative medicine teaching in four Finnish universities.

 2. The authors claim in the abstract background and in the 4 ^th^ paragraph of the introduction that none/few studies examine the outcome of palliative medicine undergraduate education. However, in recent years, there have been many studies on this such as simulation-based learning, and this can be found easily on Google Scholar e.g.

 Answer: We are grateful for this remark. When we constructed this study, there were only a few studies about this subject, and they focused on short-term impact, i.e. right after the educational intervention. However, the studies mentioned by the expert reviewer, had a slightly different focus than our study. We have added this clarification to the revised manuscript.

- Thyson, T., Schallenburger, M., Scherg, A. et al. Communication in the face of death and dying - how does the encounter with death influence the patient management competence of medical students? An outcome-evaluation. BMC Med Educ 22, 25 (2022). https://doi.org/10.1186/s12909-021-03060-5
- Boland, J. W., Brown, M. E. L., Duenas, A., Finn, G. M., & Gibbins, J. (2020). How effective is undergraduate palliative care teaching for medical students? A systematic literature review. BMJ open, 10(9), e036458. https://doi.org/10.1136/bmjopen-2019-036458
- Day, Frank C. MD, MPH; Srinivasan, Malathi MD; Der-Martirosian, Claudia PhD; Griffin, Erin PhD; Hoffman, Jerome R. MA, MD; Wilkes, Michael S. MD, PhD. A Comparison of Web-Based and Small-Group Palliative and End-of-Life Care Curricula: A Quasi-Randomized Controlled Study at One Institution. Academic Medicine 90(3):p 331-337, March 2015. | DOI: 10.1097/ACM.0000000000000607
- Weber, M., Braun, J., & Schildmann, J. (2011). Effects of a ninety-minute teaching module for fourth-year medical students on a palliative care ward with student-patient encounter. Journal of palliative medicine, 14(8), 940–944. https://doi.org/10.1089/jpm.2011.0025

3. What kind of outcomes are the authors referring to? Also, please cite the review that found 124 studies. References 12, 20-24 do not support the first sentence in the fourth paragraph of the introduction. Reference 12 was published in 2017 and is not “recent” anymore. Furthermore, reference 12 included 126 studies, not 124.  

 Answer: We are referring to long term learning outcomes. Many of the studies referred to organisation and construction of palliative medicine courses for undergraduate students. Most of the studies surveyed teachers´ comprehensions but not those of the or students or were performed in a single university. Our study provides unpublished information about national PM education in Finland.

 4. The literature gap was not well-defined. What does current research (both quantitative and qualitative) tell us about medical students’ learning experiences and attitudes on palliative care? This is the research question, and this needs to be explored more in-depth in the introduction.

 Answer: We have re-examined the studies and composed a condensed summary in the introduction. As far as we understand, the key message is that medical students improve their skills after dedicated tuition, when the outcome is measured soon after the course. However, scientific evidence on the influence of teaching on students´ attitudes is far more complicated. Both favourable and non-favourable effects on attitudes have been reported.

 5. Methods: the information on ethics should be moved to the last paragraph of the methods section as a separate section on “ethical considerations”. The first paragraph of the methods section should instead include the study design and methodology, which has not been explained. What were the eligibility criteria?

 Answer: Thank you for this comment. We have added the comment to the text (page, paragraph, line).

 6. Was the questionnaire piloted or reviewed by an expert panel before being given to the students?

 Answer: The questionnaire was constructed by a group of leading experts in palliative care teaching in Finland. It was also discussed with medical students but distributed as a pilot study. In the discussions we were convinced that the questions and issues would be well-comprehended and have clear linguistic structure. Thus, that´s, why we did not execute a pilot study.

 7. The authors included only 2 case scenarios, which are inadequate to assess the students’ palliative care knowledge. Palliative care knowledge includes many domains, such as palliative care philosophy, symptom management, and psychosocial and spiritual care. Why did the authors not use validated questionnaires such as https://doi.org/10.1089/jpm.2013.0112 and https://link.springer.com/article/10.1186/s12904-019-0430-9?

 Answer: The 2 clinical cases were included in order to assess the application of knowledge, which we considered as “skills” in this MS. Naturally, testing skills in this way would not cover the “total knowledge” comprehensively. However, management of cancer pain is included in our undergraduate curriculum as an essential content, and that is why it is such an important outcome of learning. The table 3 describes the perceived knowledge of students about PC. We agree with the Reviewer about the simplicity of our measures and that the questionnaires mentioned would be excellent, but, unfortunately, they are not available in Finnish. The next step would be translation and validation of a Finnish version of an international assessment tool. We have added this comment to the discussion (page 17, line 3).

 8. Table 3: No SD was presented, and it seems to be range in some places. Please ensure that they are in means (SD) as stated in the caption. The mean and SD seem to represent an average of all the content, and not the individual content categories. Please provide the means (SD) for each content.

 Answer:  The SDs are found at the bottom line of the tables.

 9. The question regarding whether they needed more teaching is not found in the tables.

 Answer: Thank you for this remark. This issue is mentioned in the text (page 10, lines 7-9).

 10. Discussion: “We explored students’ knowledge, skills, and attitudes according to Kirkpartrick’s four-level evaluation model of training.” I find that the questionnaire was inadequate in assessing students’ knowledge, skills, and attitudes. For knowledge, this was elaborated in a previous comment. What skills and attitudes are you referring to? In other palliative health professional education studies, skills can be assessed using skills assessment-based scores like OSCE, self-reported changes in communication skills, confidence in caring for the dying, and compassion competence etc. Attitudes can be measured using FATCOD and others. Please organize the questionnaire content in the methods “general structure of the questionnaire” to reflect this clearly.

 Answer: Thank you for this comment. We did explore the students´ attitudes in this study, but did not include the results in the original MS, because we wanted to keep the information condensed. Also, English questionnaires used detection of attitudes (such as FATCOD) are unfortunately not available in Finnish. However, over a long period of time we have developed a short questionnaire for examining attitudes towards EOL care issues, which we have also validated in this study. We have now included this tool both in the methods and in the results. Thus, the Kirkpatrick´s model has been at least partly followed. The results of the validation of our questionnaire (Factorial analysis) have been now also added as a supplemental material. We have continued to develop this tool, and have recently reported high validity and reliability of a larger version (Goyarrola et al. Pall Med Repo 2024, in press).

 11. Conclusions: “A clear association was seen between medical students’ perceived knowledge and skills.” I do not see this being reported in the results. There was no correlation analysis conducted. Knowledge was conducted using only 2 questions. The questions do not reflect skills in palliative care but rather pharmacological management (which is knowledge).

 Answer: We apologize the inaccuracy of wording. We have removed this misleading sentence. We conclude, that we have observed a connection between perceived knowledge, skills and attitudes in palliative care among students from different universities in Finland.
